# Astragalin Alleviates Neuropathic Pain by Suppressing P2X4-Mediated Signaling in the Dorsal Root Ganglia of Rats

**DOI:** 10.3389/fnins.2020.570831

**Published:** 2021-01-11

**Authors:** Mengke Wang, Xia Cai, Yueying Wang, Shizhen Li, Na Wang, Rui Sun, Jingming Xing, Shangdong Liang, Shuangmei Liu

**Affiliations:** ^1^Department of Physiology, Medical School of Nanchang University, Nanchang, China; ^2^Department of Endocrinology, Second Affiliated Hospital of Nanchang University, Nanchang, China; ^3^Undergraduate Student of the Second Clinical Department, Medical School of Nanchang University, Nanchang, China; ^4^Undergraduate Student of the Anesthesiology Department, Medical School of Nanchang University, Nanchang, China; ^5^Undergraduate Student of the Basic Medical Science Department, Medical School of Nanchang University, Nanchang, China

**Keywords:** neuropathic pain, P2X4 receptor, astragalin, chronic constriction injury, satellite glial cells, dorsal root ganglia

## Abstract

Neurologic damage often leads to neuropathic pain, for which there are no effective treatments owing to its complex pathogenesis. The purinergic receptor P2X4 is closely associated with neuropathic pain. Astragalin (AST), a compound that is used in traditional Chinese medicine, has protective effects against allergic dermatitis and neuronal injury, but its mechanism of action is not well understood. The present study investigated whether AST can alleviate neuropathic pain in a rat model established by chronic constriction injury (CCI) to the sciatic nerve. The model rats exhibited pain behavior and showed increased expression of P2X4 and the activated satellite glial cell (SGC) marker glial fibrillary acidic protein in dorsal root ganglia (DRG). AST treatment partly abrogated the upregulation of P2X4, inhibited SGC activation, and alleviated pain behavior in CCI rats; it also suppressed ATP-activated currents in HEK293 cells overexpressing P2X4. These data demonstrate that AST relieves neuropathic pain by inhibiting P2X4 and SGC activation in DRG, highlighting its therapeutic potential for clinical pain management.

## Introduction

More than 100 million people worldwide suffer from pain, which severely affects their quality of life and work productivity and imposes a considerable financial burden on their families and society (Fitzgerald and McKelvey, 2016; Murai et al., 2016; Spahr et al., 2017). Neurologic damage can lead to neuropathic pain, which includes spontaneous pain, hyperalgesia, and allodynia (sensitivity to normal and innocuous stimuli) (Gu et al., 2016). The pathogenesis of neuropathic pain is complex and poorly understood, although it is thought to involve changes in the function and structure of the central and peripheral nervous systems. The fact that conventional analgesics that target neurons are not always effective in alleviating neuropathic pain suggests that non-neurologic mechanisms may be involved.

The purinergic receptor (P2 receptor) family is divided into P2X (ionic) and P2Y (G protein-coupled) receptors (Burnstock, 2017). P2X2/3, P2X4, P2X7, P2Y1, P2Y2, and P2Y12 have been implicated in the regulation of neuropathic pain (Jarvis, 2010; Tsuda, 2017). P2X4 was shown to be upregulated in the ipsilateral spinal dorsal horn following peripheral nerve injury (Tsuda et al., 2003), and P2X4 knockout mice were insensitive to pain induced by peripheral nerve injury (Ulmann et al., 2008). Conversely, intrathecal injection of P2X4-positive cultured microglial cells into normal rodents enhanced their response to painful tactile stimulation (Tsuda et al., 2009a). These studies indicate that P2X4 is closely associated with neuropathic pain.

P2X4 expressed by satellite glial cells (SGC) of the dorsal root ganglia (DRG) has been shown to be involved in neuropathic pain (Ying et al., 2017; Yuan et al., 2018; Yang et al., 2019). Large amounts of ATP are released after nerve injury, activating P2X4 on SGCs (Kushnir et al., 2011; Kobayashi et al., 2013; Ying et al., 2017). Chronic constriction injury (CCI) to the sciatic nerve triggers the release of inflammatory factors and induces upregulation of the activated SGC marker glial fibrillary acidic protein (GFAP), which is implicated in the modulation of neuropathic pain (Yuan et al., 2018). Astragalin (AST) is a flavonoid extracted from the white stamen of some flowers with known pharmacologic properties including anti-inflammatory, antioxidant, and anti-allergic activities and protective effects against dermatitis and neuronal injury (Rey et al., 2019). In the present study, we investigated whether AST also has analgesic effects using a rat model of CCI-induced neuropathic pain.

## Materials and Methods

### Animals

Male Sprague–Dawley rats weighing 200 ± 20 g were purchased from Changsha Tianqin Biological Co. (Hunan, China) and allowed to acclimate to the laboratory environment for 7 days before they were used in experiments. The rats were housed in a ventilated room with the temperature controlled at 23°C on a 12:12 h light/dark cycle with free access to water and food. All animal procedures were approved by the Animal Protection and Use Committee of the Medical School of Nanchang University. The experiments in this study followed International Association for the Study of Pain ethics guidelines and were carried out according to the guidelines of Animal Research: Reporting of *In Vivo* Experiments.

### Drugs

Astragalin (C_21_H_20_O_11_, MW 448.38, >98% purity) was purchased from Nanjing Herb Source Biotechnology Co. (Jiangsu, China) and dissolved in 0.5% dimethyl sulfoxide (DMSO). The P2X4 antagonist 5-(3-bromophenyl)-1H-benzofuro(3,2-e)(1,4)diazepin-2(3H)-one (5-BDBD) was purchased from Bio-Techne (Shanghai, China; cat. no. 3579). All other chemicals were of analytic grade and obtained from standard commercial suppliers.

### Experimental Design

In order to determine the concentration of AST, we carried out a preliminary experiment and designed three concentrations (25, 50, and 100 mg/kg) of AST to observe the analgesic effect of AST by intragastric administration. The dose of AST was selected based on the results of our pilot experiments and previous literature (Zheng et al., 2019). Rats were randomly divided into six groups (*n* = 8): control (Ctrl), sham operation (Sham), rats with sciatic nerve CCI (CCI), Ctrl rats treated with AST (Ctrl + AST), CCI rats treated with AST (CCI + AST), and DMSO-treated CCI rats (CCI + DMSO). The control rats were designed as a blank control without any drugs. The CCI + AST and Ctrl + AST groups were intragastrically administered with AST (50 mg/kg/day, dissolved into 10 mg/ml concentration with 0.5% DMSO, 1 ml volume for 200 g rats) once a day for 2 weeks starting on day 2 after CCI. Rats in the CCI + DMSO group were orally administered by gavage with the same volume of DMSO (0.5%, dissolved in normal saline) daily for 2 weeks. In order to evaluate the role of P2X4 receptors and the involvement of extracellular signal-regulated kinase (ERK) signaling in the effects of P2X4, the P2X4 antagonist 5-BDBD was added in the experiment. 5-BDBD (dissolved in 0.5% DMSO) was prepared to be 100 μM. After fixation under anesthesia, each rat in the CCI + 5-BDBD group was intravenously injected with 10 μl of 5-BDBD through the tail vein, while the CCI + DMSO group rat was injected with 10 μl of 0.5% DMSO once daily from days 7 to 13 after CCI operation. A new microsyringe was changed every day for each rat. There were no unexpected, new, and/or significant hazards or risks associated with this work.

### Animal Model

We used CCI of the rat sciatic nerve as a neuropathic pain model. Rats were anesthetized by intraperitoneal injection of 10% chloral hydrate (3 ml/kg). After immobilizing the rat in a prone position, the right hind leg was disinfected with 75% ethanol solution. A skin incision was made along the inside of the femur, and the sciatic nerve was completely exposed after separating the subcutaneous tissue and muscle. The sciatic nerve was ligated four times using a 4-0 chromic gut suture starting 7 mm distal to the sciatic nerve trunk, with each suture separated by a distance of 1 mm. Sham rats underwent the same operation as CCI rats but without nerve ligation.

### Behavioral Tests

Pain behavior was measured on day 0 (before the experiment) and days 1, 3, 5, 7, 9, 11, 13, and 15 after CCI. Rats were allowed to adapt to the surrounding environment before behavioral testing. The mechanical withdrawal threshold (MWT) was determined by observing the withdrawal response induced by mechanical stimulation using a BME-404 electronic mechanical pain detector (Tsuda et al., 2009b; Leung and Cahill, 2010). The rat was placed on a metal frame with a 1 × 1-mm mesh and allowed to move freely during the 15-min adaptation period. The right hind foot of the rat was stimulated five times at 5-min intervals using the metal wire of the pain detector through the mesh from bottom to top. Average stimulus intensity causing foot withdrawal of the five times was used as the MWT(g) in rats.

Thermal withdrawal latency (TWL) was assessed with a BME-410C Thermal Paw Stimulation System (Boerni, Tianjin, China) (Wang et al., 2016; Li et al., 2017; Peng et al., 2017). The rat was placed on a 3-mm-thick plexiglass plate and allowed to adapt to the environment for 15 min at room temperature (26.0 ± 0.5°C). When the hind paw on the side of CCL contacted the glass plate, a light from the stimulator focused on the palm. The latency from the start of irradiation to hind limb retraction was recorded. Five trials were performed for each rat at 5-min intervals, and the average measurement was taken as TWL. Irradiation time never exceeded 30 s to avoid thermal injury. The experimenter who carried out the behavioral tests was blinded to the treatments of the animals.

### Molecular Docking

Using human (h) P2X4 as the target protein and AST as the ligand, molecular docking simulations were carried out using the AutoDock Tools (ADT) of AutoDock 4.2 software^1^ and the Python scripts ligand4.py and prepare receptor4.py. The target protein binding pocket was identified with the ADT molecular observer. The parameters were saved as system default values. Finally, MGLTools^2^ and PyMOL software^3^ were used to view the output file.

### Double Immunofluorescence Labeling of P2X4 and GFAP

L4–L6 DRG were removed from four rats in each group anesthetized with chloral hydrate on day 15 after CCI and fixed with 4% paraformaldehyde in phosphate-buffered saline (PBS) for 1 day (Burnstock, 2013; Jia et al., 2017), followed by dehydration overnight at 4°C in 20% sucrose solution. The tissue was cut into sections at a thickness of 10 mm on a cryostat (Leica Biosystems, Wetzlar, Germany); the sections were washed three times for 5 min each in PBS, then permeabilized in 0.3% Triton X-100 for 15 min. Non-specific antigens were blocked with 10% goat serum (Zhongshan Golden Bridge Company, Zhongshan, China) for 1 h at 37°C. Sections were incubated overnight at 4°C with mouse anti-GFAP (1:200 dilution; 38014, Signalway Antibody, Nanjing, China) and rabbit anti-P2X4 (1:200; APR-002, Alomone Labs, Jerusalem, Israel) primary antibodies and washed three times for 10 min each in PBS (Yuan et al., 2018). They were then incubated with secondary antibodies conjugated with fluorescein isothiocyanate or tetramethylrhodamine (1:200 dilution) for 1 h at 37°C, followed by three 10-min washes in PBS. Immunoreactivity was visualized with an epifluorescence microscope (Olympus, Tokyo, Japan). Eight different fields of vision from different DRGs were selected to take photos. The fluorescence intensity values of the eight photos were measured by ImageJ software. Eight data were obtained from each group. Then, the data of each group were analyzed statistically.

### Real-Time Quantitative (q)PCR

L4–L6 DRG isolated from rats anesthetized with chloral hydrate on day 15 after CCI were rinsed with ice-cold PBS, and total RNA was extracted using the Total RNA Extraction kit (Promega Biotech, Shanghai, China) according to the manufacturer’s instructions. The RNA was reverse transcribed into cDNA using the cDNA Synthesis kit (Thermo Fisher Scientific, Shanghai, China). RNA samples were analyzed by qPCR with SYBR Green MasterMix on an ABI Prism 7500 instrument (Applied Biosystems, Foster City, CA, United States) using the following forward and reverse primers (Sangon Biotech, Shanghai, China): P2X4, 5′-CCCTTTGCCTGCCCAGATAT-3′ and 5′-CCGTACGCCTTGGTGAGTGT-3′; and β-actin, 5′-AAGATCCTGACCGAGCGTGG-3′ and 5′-CAGCACTGTGTTGGCATAGAGG-3′. The threshold cycle (CT) was used to calculate the expression level of target genes in each sample normalized to that of β-actin (2^–Δ^ ^Δ^ ^*CT*^ method).

### Western Blotting

On day 15 after CCI, L4–L6 DRG was isolated from rats anesthetized with chloral hydrate and immediately washed with precooled PBS. DRG tissue was homogenized on ice by trituration in lysis buffer [50 mM TrisCl (pH 8.0), 150 mM NaCl, 0.1% dodecyl sodium sulfate, 1% Nonidet P-40, 0.5% sodium deoxycholate, 100 μg/ml phenyl methylsulfonyl fluoride, and 1 μg/ml aprotinin] and incubated on ice for 40 min. The homogenate was centrifuged at 6,000 × *g* for 10 min, and the supernatant was collected. Total protein concentration in the supernatant was determined with the Lowry method. After dilution with sample buffer [100 mM TrisCl, 200 mM dithiothreitol, 4% sodium dodecyl sulfate (SDS), 0.2% bromophenol blue, and 20% glycerol] and heating at 95°C for 10 min, samples containing equal amounts of protein (20 μg) were separated by SDS–polyacrylamide gel electrophoresis (Bio-Rad, Hercules, CA, United States) on a 12% acrylamide gel, and the proteins were transferred to a polyvinylidene difluoride membrane. Non-specific antigens were blocked by incubating the membrane for 2 h at room temperature in 5% skim milk; this was followed by overnight incubation at 4°C with the following primary antibodies: rabbit anti-P2X4 (1:500; Alomone Labs), rabbit anti-ERK1/2 (1:1,000), and anti-phosphorylated (p)ERK1/2 (1:2,000) (both from Cell Signaling Technology, Danvers, MA, United States); and rabbit anti-tumor necrosis factor receptor (TNF-R)1 (1:800) and mouse anti-GFAP (1:800) (both from Abcam, Cambridge, MA, United States). After incubation with horseradish peroxidase-conjugated secondary antibody for 2 h, protein bands were visualized with a chemiluminescent substrate (Thermo Fisher Scientific, Waltham, MA, United States). The membrane was stripped and reprobed with anti-β-actin antibody (1:800; Abcam), which was used to normalize target protein expression level. Quantification of protein level expressed as integrated optical density was measured using Image Pro-Plus software (Media Cybernetics, Rockville, MD, United States).

### Cell Culture and Transfection

HEK293 cells were grown in Dulbecco’s modified Eagle’s medium containing 10% fetal bovine serum, 1% penicillin, and streptomycin in a 37°C, 5% CO_2_ humidified incubator. Full-length cDNA encoding rat P2X4 (1,185 bp) was inserted into the pcDNA3.1(+)/EGFP/IRES vector (ampicillin resistance) containing an enhanced green fluorescent protein (EGFP) reporter to generate pcDNA3.1(+)/EGFP/IRES-P2RX4. Cloning site was *Nhe*I/*Xba*I. The plasmid was constructed by Shanghai Nuovo Biological Technology Co. (Shanghai, China) and was transiently transfected into cells using Lipofectamine 2000 reagent (Invitrogen, United States) according to the manufacturer’s instructions. When the cells reached 70–80% confluence, the culture medium was replaced with OptiMEM for 2 h. The transfection medium was prepared as follows: (a) Dilute 4 μg of plasmid with OptiEME to 250 μl, (b) dilute 10 μl of Lipo2000 with OptiEME to 250 μl, and (c) mix the above two solutions and incubate at room temperature for 20 min. Then, 500 μl of cDNA/lipofectamine solution was added to each well followed by incubation for 6 h at 37°C and 5% CO_2_. Control cells were transfected with the empty pcDNA3.1(+)/EGFP/IRES vector. After incubation, the cells were washed in minimal essential medium containing 10% fetal bovine serum and cultured for 24–48 h. GFP fluorescence was evaluated to determine the transfection efficiency. Whole-cell patch clamp recordings were carried out 1–2 days after transfection.

### Electrophysiology

Electrophysiologic recordings were carried using a whole-cell patch clamp amplifier (Axopatch 200B; Axon Instruments/Molecular Devices, San Jose, CA, United States). HEK293 cells with green fluorescence (i.e., expressing P2X4) were selected for recording. Microelectrodes were filled with an internal solution composed of (mM) K gluconate (145), MgATP (2), EGTA (0.75), HEPES (10), CaCl_2_ (0.1), and Na_3_GTP (0.3). The bath was continuously perfused with extracellular solution composed of (mM) KCl (2.5), NaCl (126), glucose (10), CaCl_2_ (2.4), MgCl_2_ (1.2), and NaHCO_3_ (18). The osmotic pressure of the extracellular and internal solutions was adjusted to 340 mOsm with sucrose. The pH of the extracellular solution was adjusted to 7.4 with NaOH and that of the internal solution was adjusted to 7.3 with KOH. The resistance of the recording electrode was 2–6 MΩ. The holding potential was −70 mV. ATP (Sigma-Aldrich, St. Louis, MO, United States) and the P2X4 antagonist 5-BDBD were dissolved in extracellular solution; AST was dissolved in 0.5% DMSO and diluted to different concentrations with extracellular solution. The drugs were rapidly administered through a manifold consisting of 10 capillaries made of fused silica coated with polyimide with an inner diameter of 200 μm. The distance from the tubule mouth to the examined cell was approximately 100 μm. The solution was transported from a separate container by gravity flow. One barrel was used to apply the drug-free extracellular solution to rapidly terminate drug application. The drug was applied every 4 min for 2 s, and the effect was reproducible. The data were low-pass filtered at 2 Hz, digitized at 5 kHz, and stored on a laboratory computer using the Digidata 1200 system and pClamp 10.0 software (Axon Instruments/Molecular Devices).

### Statistical Analysis

Data were analyzed using SPSS software (SPSS Inc., Chicago, IL, United States) and are presented as mean ± SEM. Multiple comparisons for western blotting, qPCR, immunofluorescence, and electrophysiology data were performed by one-way analysis of variance (ANOVA) followed by Fisher’s *post hoc* test. Pain behavior data were evaluated by two-way ANOVA. *p* < 0.05 was considered statistically significant.

## Results

### AST Alleviates Pain Behavior in CCI Rats

**FIGURE 1:**
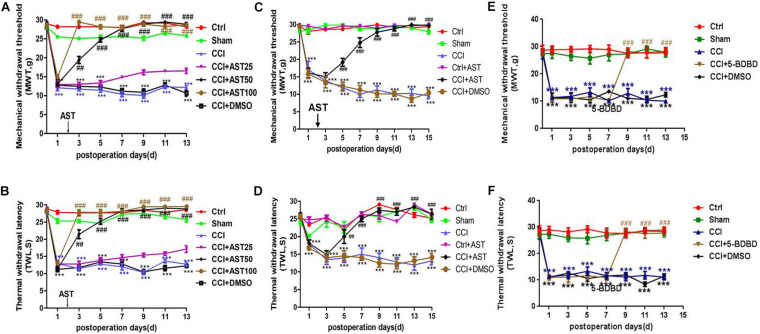
Astragalin (AST) alleviates pain behavior in chronic constriction injury (CCI) rats. **(A,B)** Different dose of AST treatment CCI rats increased the mechanical withdrawal threshold (MWT) **(A)** and thermal withdrawal latency (TWL) **(B)**. The 50- and 100-dose groups significantly reduced the pain threshold of CCI rats, and there was no difference between them. The 25-dose group had a weak effect on pain behavior. **(C,D)** On the indicated days after CCI, MWT **(C)** and TWL **(D)** were significantly reduced relative to sham-operated (Sham) rats. Treatment with AST increased MWT and TWL from day 5 to 15. **(E,F)** On the indicated days after CCI, MWT **(E)** and TWL **(F)** were significantly reduced relative to sham-operated (Sham) rats. Treatment with 5-(3-bromophenyl)-1H-benzofuro(3,2-e)(1,4)diazepin-2(3H) -one (5-BDBD) increased MWT and TWL from day 9 to 13. Data are presented as mean ± SEM (*n* = 8 rats/group). ***p < 0.001 vs Ctrl; ^##^*p* < 0.01, ^###^*p* < 0.001 vs CCI.

The preliminary experiment results showed that a different dose of AST treatment CCI rats increased the MWT (Figure 1A) and TWL (Figure 1B). Dose groups (50 and 100 mg/kg) significantly reduced the pain threshold of CCI rats, and there was no difference between them. One dose group (25 mg/kg) had a weak effect on pain behavior. According to the above result and the literature (Zheng et al., 2019), we chose 50 mg/kg by intragastric administration for follow-up experiments. MWT and TWL were measured before the operation and no differences were observed across groups (*p* > 0.05). On days 1, 3, 5, 7, 9, 11, 13, and 15 post-CCI, MWT (Figure 1C) and TWL (Figure 1D) were significantly lower in the CCI rats than in the Ctrl group (*p* < 0.001) and were increased in the CCI + AST group from day 5 to day 15. On the final day of the experiment, MWT (Figure 1C) declined in the CCI (9.238 ± 0.774) and CCI + DMSO (10.04 ± 0.783) groups compared to the Ctrl group (29.219 ± 0.28), while an increase was observed in CCI + AST rats (29.691 ± 0.164). There were no differences between the Ctrl, Sham (27.998 ± 0.528), and Ctrl + AST groups (29.891 ± 0.086) (*p* > 0.05), and no difference between CCI and CCI + DMSO groups (*p* > 0.05). By day 15, TWL was decreased in CCI (13.118 ± 1.103) and CCI + DMSO (14.13 ± 1.105) groups relative to the Ctrl group (25.181 ± 0.62) but increased in CCI + AST rats (26.975 ± 0.993) (Figure 1D). There were no differences between Ctrl, Sham (24.684 ± 0.685), and Ctrl + AST (26.359 ± 0.331) groups (*p* > 0.05), or between CCI and CCI + DMSO (*p* > 0.05) groups. 5-BDBD increased MWT and TWL from day 9 to day 13 compared with CCI rats (Figures 1E,F).

### Molecular Docking of AST With hP2X4 Protein

**FIGURE 2:**
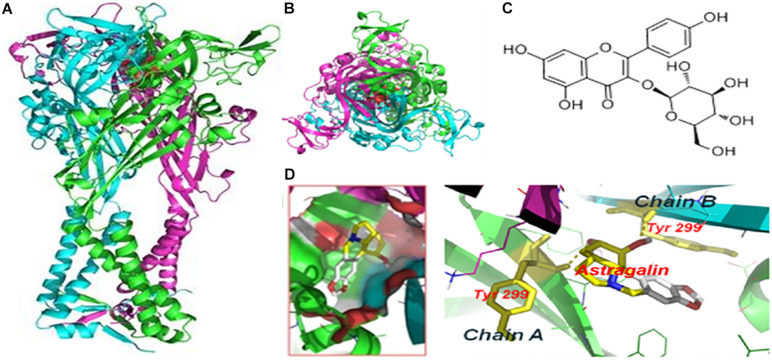
Molecular docking of AST with hP2X4 protein. **(A)** Forward view. **(B)** Top view. **(C)** Chemical structure of AST. **(D)** Docking bag. The green and blue rod structures are the A and B chains of the hP2X4 protein, respectively; the yellow dotted line is the hydrogen bond connecting the residue on the chain and AST. The binding energy of AST with P2X4 was –7.3 kcal/mol.

We performed molecular docking to predict whether AST and P2X4 interact. The optimal docking configuration between AST and hP2X4 revealed that AST strongly interacts with Tyr299 in the A and B chains of hP2X4 protein. The binding energy for the interaction was calculated as −7.3 kcal/mol (Figure 2).

### AST Decreases P2X4 Level in DRG of CCI Rats

**FIGURE 3:**
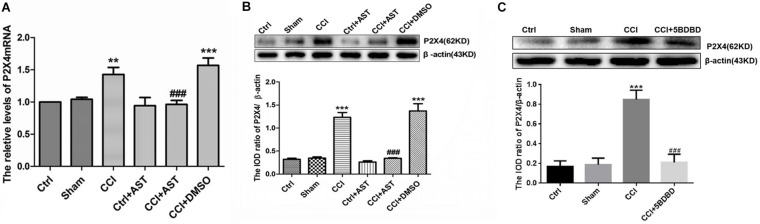
AST decreases P2X4 level in the dorsal root ganglia (DRG) of CCI rats. **(A,B)** Relative expression of P2X4 mRNA **(A)** and protein **(B)** was increased in CCI rats compared to the Ctrl group, and decreased in AST-treated CCI rats compared to CCI rats. **(C)** P2X4 protein expression in CCI rats was decreased by P2X4-selective antagonist 5-BDBD compared with that in the untreated CCI rats. Data are presented as mean ± SEM of three independent experiments for each bar (Each group sample was from eight rats.). **p < 0.01, ***p < 0.001 vs Ctrl, ^###^*p* < 0.001 vs CCI.

The effect of AST on P2X4 mRNA and protein levels in DRG of CCI rats was examined by real-time qPCR and western blotting, respectively. P2X4 mRNA level was elevated in CCI rats (1.43 ± 0.11) relative to the Ctrl group (*p* < 0.01) but was reduced in CCI + AST rats (0.96 ± 0.06) relative to CCI rats (*p* < 0.001) (Figure 3A). There were no significant differences in *P2*X*4* transcript level between the Ctrl, Sham (1.04 ± 0.33), and Ctrl + AST (0.94 ± 0.13) groups or between CCI + DMSO (1.57 ± 0.11) and CCI (*p* > 0.05) groups. P2X4 protein level was also higher in CCI rats (1.23 ± 0.11) than in Ctrl rats (0.32 ± 0.02) (*p* < 0.001), and lower in the CCI + AST group (0.34 ± 0.01) than in the CCI group (*p* < 0.001) (Figure 3B). There were no differences between the Ctrl, Sham (0.35 ± 0.03), and Ctrl + AST (0.26 ± 0.03) groups or between the CCI + DMSO (1.37 ± 0.16) and CCI groups (*p* > 0.05). In order to observe whether blocking the activation of P2X4 receptor affects the expression of P2X4 receptor, we also detected the expression of P2X4 receptor in CCI rats treated with 5-BDBD. The results were as follows: Ctrl (0.17 ± 0.03), Sham (0.188 ± 0.04), CCI (0.85 ± 0.06), and CCI + 5-BDBD (0.21 ± 0.05) (Figure 3C). P2X4 protein expression in CCI rats was also decreased by 5-BDBD compared with that in the untreated CCI rats. Thus, AST treatment as well as 5-BDBD induced the downregulation of P2X4 in the DRG of CCI rats.

### AST Inhibits SGC Activation in DRG of CCI Rats

**FIGURE 4:**
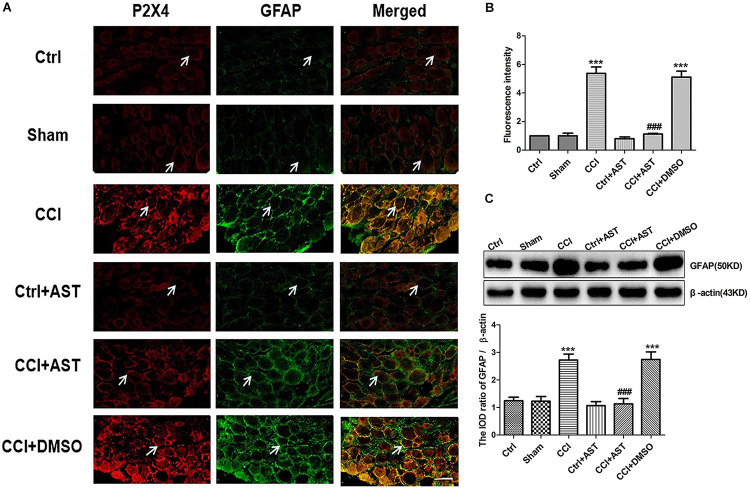
AST inhibits satellite glial cell (SGC) activation in DRG of CCI rats. **(A)** Colocalization of P2X4 and GFAP in rat DRG was detected by double immunofluorescence labeling. Green and red signals are GFAP and P2X4 labeled with fluorescein isothiocyanate and tetramethylrhodamine, respectively. Arrows indicate cells positive for both P2X4 and GFAP. Scale bar, 20 μm. **(B)** P2X4 and GFAP colocalization was more extensive in CCI rats than in Ctrl rats and was lower in CCI rats treated with AST than in untreated CCI rats. **(C)** Western blot analysis of GFAP protein level. GFAP expression was increased in CCI rats compared to Ctrl rats, an effect that was abrogated by AST treatment. Data are presented as mean ± SEM. For fluorescence analysis, eight photos from four rats were taken from each group to get the mean number. For western blotting analysis, each group samples were from eight rats, and three independent repeated experiments were conducted. ***p < 0.001 vs Ctrl, ^###^*p* < 0.001 vs CCI.

We examined whether P2X4 is expressed in activated SGCs by double immunofluorescence labeling of DRG with antibodies against P2X4 and GFAP. The results showed that P2X4 colocalized with GFAP in SGCs (Figure 4A), with greater colocalization in the CCI group (5.38 ± 0.45) than in the Ctrl group (1.0 ± 0.12) (*p* < 0.001) and less colocalization in the CCI + AST group (1.13 ± 0.04) than in CCI rats (Figure 4B). There were no differences in P2X4 and GFAP colocalization between the Ctrl, Sham (1.00 ± 0.18), and Ctrl + AST (0.8 ± 0.12) groups or between the CCI + DMSO (5.11 ± 0.41) and CCI groups (*p* > 0.05).

To determine the degree of SGC activation, we also evaluated GFAP protein expression in DRG by western blotting (Figure 4C). GFAP expression was upregulated in CCI rats (2.73 ± 0.22) compared to Ctrl rats (1.24 ± 0.13) (*p* < 0.001). Meanwhile, no differences were observed between Sham (1.22 ± 0.17), Ctrl, and Ctrl + AST (1.07 ± 0.14) groups (*p* > 0.05). GFAP level was lower in CCI + AST rats (1.13 ± 0.20) than in CCI rats (*p* < 0.001). There was no difference in GFAP protein level between the CCI + DMSO (2.74 ± 0.28) and CCI groups (*p* > 0.05).

### AST Inhibits TNF-R1 Expression in DRG of CCI Rats

**FIGURE 5:**
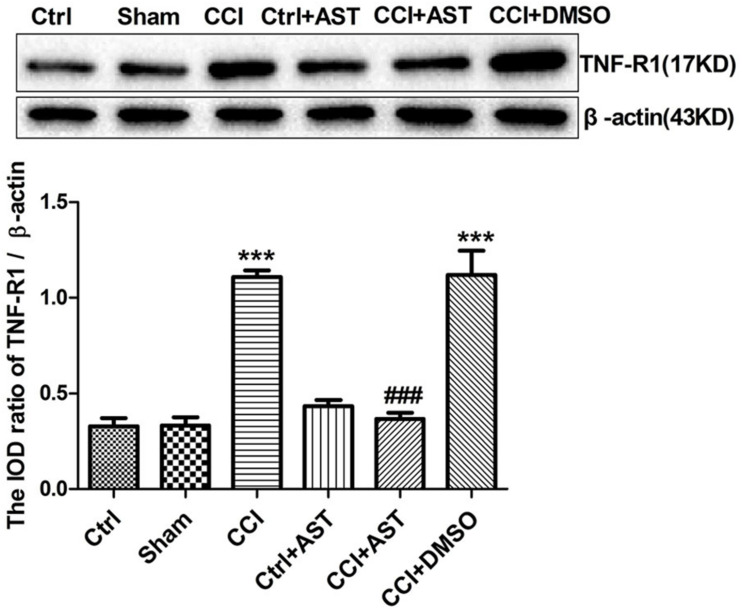
AST inhibits TNF-R1 expression in the DRG of CCI rats. TNF-R1 was upregulated in CCI rats relative to Ctrl rats and downregulated by AST treatment compared to untreated CCI rats. Data are presented as mean ± SEM of three independent experiments for each bar (Each group sample was from eight rats.). ***p < 0.001 vs Ctrl, ^###^*p* < 0.001vs CCI.

TNF-α released from the activated glial cells acts on TNF-R1 expressed by DRG neurons. Upregulation of TNF-R1 expression in DRG neurons enhances their sensitivity to mechanical and thermal stimuli following peripheral nerve injury. Western blotting analysis revealed increased TNF-R1 expression in the CCI group (1.11 ± 0.03) compared to the Ctrl group (0.33 ± 0.04) (*p* < 0.001; Figure 5). TNF-R1 level was lower in the CCI + AST group (0.37 ± 0.03) than in the CCI group (*p* < 0.001); however, there were no differences between the Ctrl, Sham (0.33 ± 0.04), and Ctrl + AST (0.43 ± 0.03) groups or between the CCI + DMSO (1.12 ± 0.13) and CCI groups (*p* > 0.05).

### AST Inhibits ERK Signaling in DRG of CCI Rats

**FIGURE 6:**
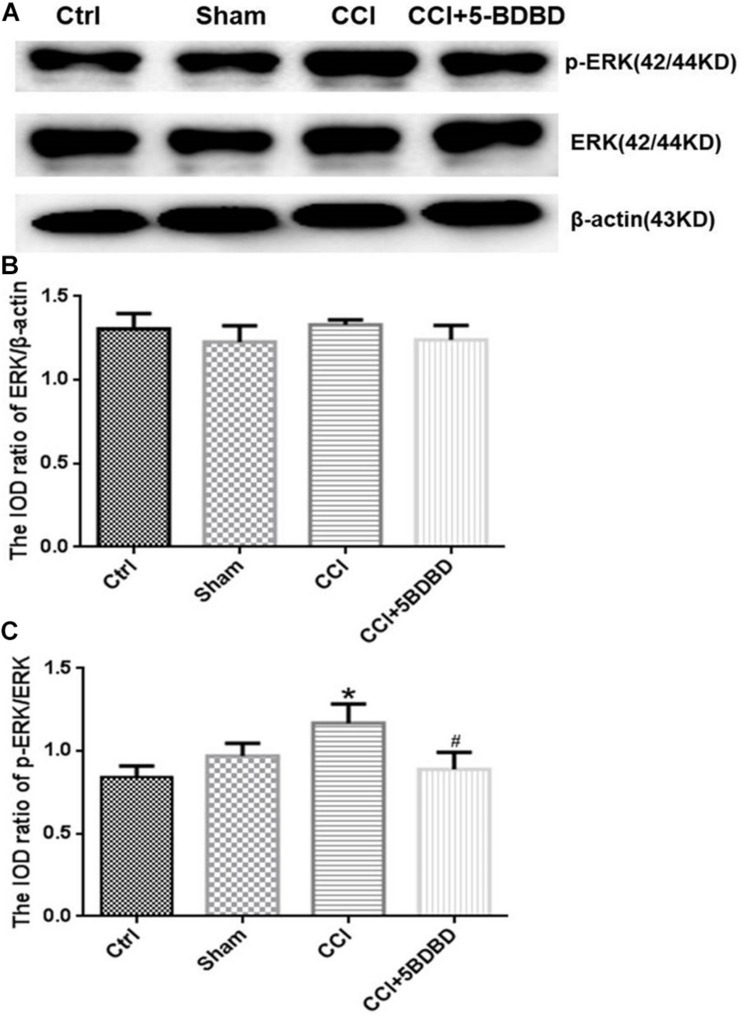
5-BDBD inhibits extracellular signal-regulated kinase (ERK) signaling in DRG of CCI rats. **(A)** Western blot analysis of pERK, ERK, and β-actin levels. **(B)** Total ERK level did not differ significantly between groups. **(C)** pERK1/2 level was increased in CCI rats compared to Ctrl rats and decreased in CCI rats by treatment with the P2X4 antagonist 5-BDBD. Data are presented as mean ± SEM of three independent experiments for each bar (Each group sample was from eight rats.) *p < 0.05 vs Ctrl, ^#^*p* < 0.05 vs CCI.

**FIGURE 7:**
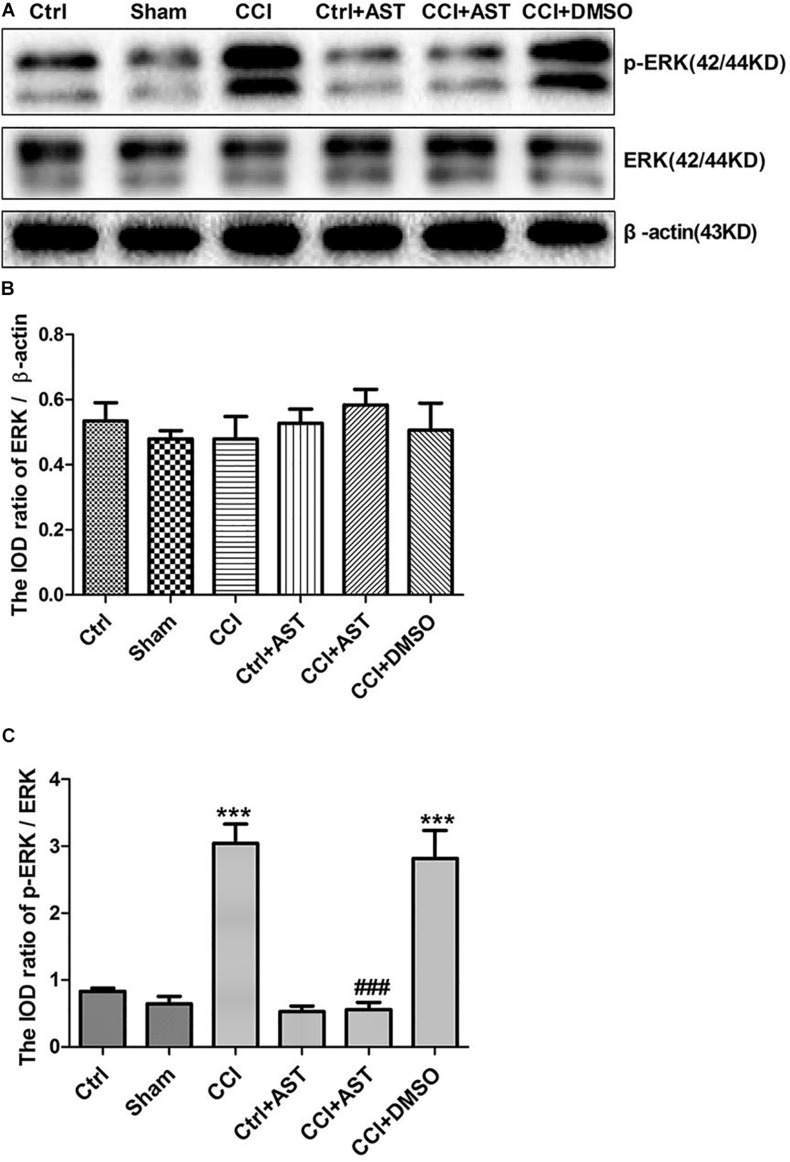
AST inhibits ERK1/2 activation in the DRG of CCI rats. **(A)** Western blot analysis of pERK, ERK, and β-actin levels. **(B)** Total ERK level did not differ significantly between groups. **(C)** pERK1/2 level was increased in CCI rats compared to Ctrl rats and decreased in CCI rats by AST treatment. Data are presented as mean ± SEM of three independent experiments for each bar (Each group sample was from eight rats.). ***p < 0.001 vs Ctrl, ^###^*p* < 0.001 vs CCI.

To determine whether the ERK signaling pathway mediates the effects of P2X4, we examined the expression levels of ERK and phosphorylated extracellular-regulated protein kinase (pERK) in DRG. Total ERK level did not differ significantly across groups (*p* > 0.05; Figures 6A,B, 7A,B). The increase in pERK1/2 level in the CCI group was abrogated by treatment with the P2X4 antagonist 5-BDBD (0.88 ± 0.10) relative to the Ctrl group (1.16 ± 0.11) (*p* < 0.001; Figures 6A,C). pERK1/2 level was significantly higher in CCI rats (3.04 ± 0.29) than in Ctrl rats (0.82 ± 0.25) (*p* < 0.001; Figures 7A,C), but was lower in CCI + AST rats (0.55 ± 0.11) than in CCI rats (*p* < 0.001). There were no differences in pERK1/2 between the Ctrl, Sham (0.65 ± 0.1), and Ctrl + AST (0.53 ± 0.09) groups or between the CCI + DMSO (2.82 ± 0.42) and CCI groups (*p* > 0.05).

### Effect of AST on ATP-Activated Currents in HEK293 Cells Expressing P2X4

**FIGURE 8:**
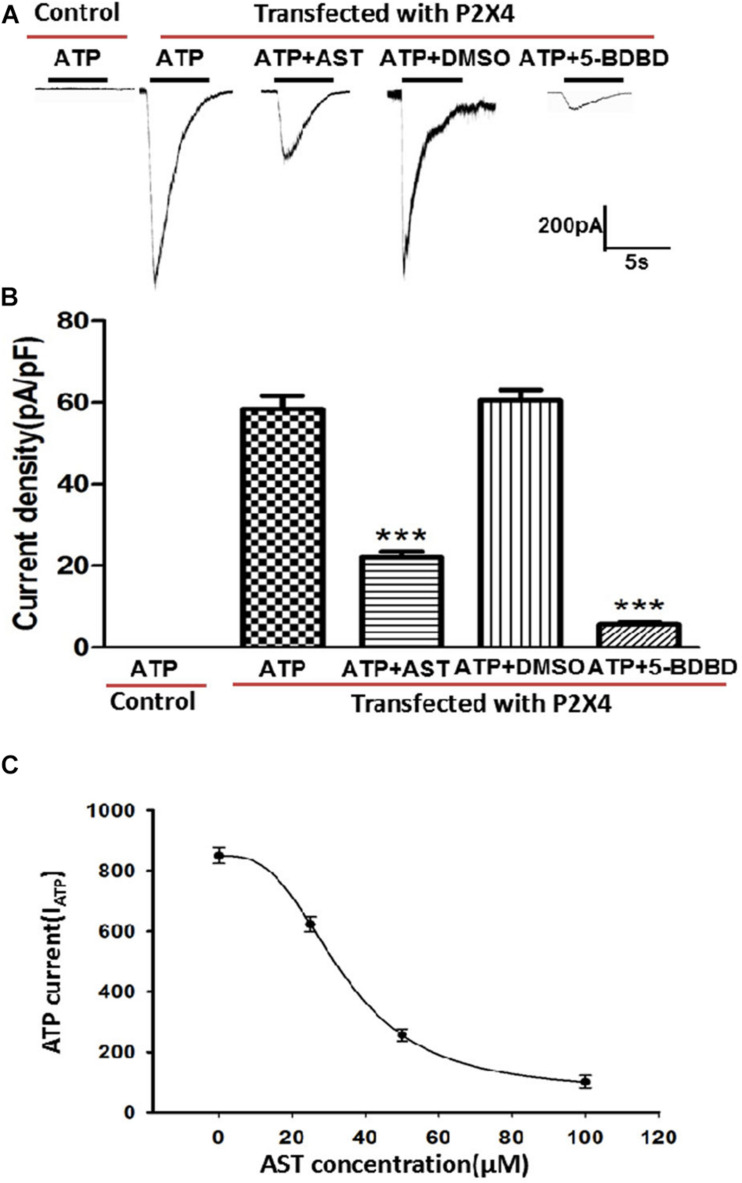
Effect of AST on ATP-activated currents in HEK293 cells expressing P2X4. **(A)** Representative traces showing that AST (50 μM) inhibited the ATP (100 μM)-induced current in HEK293 cells transfected with P2X4 plasmid. Control cells not transfected with P2X4 do not respond to ATP at the concentrations. **(B)** Histogram of current density in the presence of indicated drugs. AST (50 μM) and the P2X4-selective antagonist 5-BDBD (10 μM) inhibited the ATP-activated current in HEK293 cells. DMSO has no effect on ATP-activated current. **(C)** Concentration–response curve for AST obtained with 100 μM ATP. The IC_50_ value 33.73 ± 2.25 μM was derived from the equation of the sigmoidal function giving the best fit to the data. Each data point represents mean ± SEM of six cells. ***p < 0.001 compared with ATP current produced by the cells transfected with P2X4 receptor in the absence of drugs.

ATP-activated currents in HEK293 cells transfected with pcDNA3.1(+)/EGFP/IRES-P2RX4 plasmid and control cells transfected with the empty pcDNA3.1(+)/EGFP/IRES vector were recorded by whole-cell patch clamping. Currents were generated in HEK293 cells overexpressing P2X4 but not in control cells by application of ATP (100 μM). AST (50 μM) treatment blocked the ATP current generated in P2X4-overexpressing cells, but to a lesser degree than the P2X4 antagonist 5-BDBD (10 μM). DMSO has no inhibition effect on ATP-activated current in cells expressing P2X4 receptors. AST inhibited ATP activated current in a manner of concentration–response (Figure 8).

## Discussion

Neuropathic pain is a type of chronic pain caused by nerve injury (Gu et al., 2016). The results of the present study demonstrate that the pain threshold of CCI rats was significantly lower than that of Ctrl rats; in contrast, there was no hyperalgesia in the Sham group, indicating that the neuropathic pain model was successfully established. AST is a compound used in traditional Chinese medicine that has anti-inflammatory and neuroprotective effects (Rey et al., 2019). We found here that MWT and TWL were increased in AST-treated and 5-BDBD-treated CCI rats compared to untreated CCI rats, indicating that P2X4 receptor is involved in pain transmission, and AST can alleviate mechanical and thermal hyperalgesia.

P2X4 expression is upregulated in peripheral nerve injury, and P2X4 activation in DRG is a key factor in the development and maintenance of neuropathic pain (Kushnir et al., 2011; Burnstock, 2013; Kobayashi et al., 2013; Ying et al., 2017). Using a molecular docking approach, we determined that AST can interact with P2X4. Previous studies have suggested that inhibiting P2X4 is a potential strategy for the management of neuropathic pain (Burnstock, 2013; Stokes et al., 2017). Our result also showed that 5-BDBD treatment in CCI rats decreased the expression of P2X4 and alleviated the pain behavior. It suggests that P2X4 was implicated in the neuropathic pain. To determine whether AST exerts its analgesic effect via P2X4 activation, we examined P2X4 mRNA and protein expression in DRG and found that both were upregulated in CCI rats relative to sham-operated rats; AST treatment reversed this trend, demonstrating that AST inhibits P2X4-mediated signaling to relieve neuropathic pain in a rat model. This was supported by our whole-cell patch clamping experiments using HEK293 cells overexpressing hP2X4; the ATP-activated current in these cells was reduced by treatment with AST or the selective P2X4 antagonist 5-BDBD.

Primary sensory ganglia contain sensory neurons and surrounding SGCs (Hanani, 2005), which have been implicated in the transmission of pain information (Huang et al., 2013; Rollini et al., 2017). P2X4 is mainly expressed in SGCs of DRG (Kushnir et al., 2011; Kobayashi et al., 2013). In this study, we observed that P2X4 colocalized with GFAP in SGCs to a greater extent in CCI rats than in Ctrl rats. GFAP is upregulated in activated SGCs (Hanani, 2005; Burnstock, 2013; Costa and Moreira, 2015). In the present study, SGCs were activated in CCI rats; treatment with AST reduced P2X4 and GFAP colocalization in SGCs relative to CCI rats. This provides additional evidence that AST suppresses the transmission of nociceptive signals by decreasing P2X4 expression in DRG SGCs, thereby inhibiting SGC activation to alleviate neuropathic pain.

Neuropathic pain involves communication between SGCs and neurons in sensory ganglia (Hanani, 2005; Huang et al., 2013). Activated glial cells release the proinflammatory cytokine TNF-α, which acts on TNF-R1 expressed by DRG neurons, enhancing their sensitivity to mechanical and thermal stimuli following peripheral nerve injury (Trang and Salter, 2012; Tsuda et al., 2013). We observed that TNF-R1 was upregulated in DRG of CCI rats. We speculate that the suppression of P2X4 expression and SGC activation by AST in CCI rats inhibits the release of TNF-α by these cells, leading to downregulation of TNF-R1 in neurons and a reduction in pain sensitivity.

The ERK signaling pathway in glial cells and neurons is important for the transmission of pain information in the CCI model (Zhao et al., 2018). P2X4 is known to activate ERK signaling (Ulmann et al., 2008; Tsuda et al., 2009a; Leung and Cahill, 2010), which stimulates the production of cytokines involved in the intensification of neuropathic pain sensation (Popiolek-Barczyk et al., 2015; Rojewska et al., 2016). In this study, ERK phosphorylation was significantly increased after CCI, consistent with a previous report (Jurga et al., 2017). This suggests that ERK1/2 signaling functions downstream of P2X4 to mediate neuropathic pain, which is supported by our observation that administration of 5-BDBD decreased pERK1/2 level in CCI rats. AST decreased P2X4 expression and pERK levels in DRG of CCI rats. These results suggest that AST may alleviate CCI-induced neuropathic pain by decreasing the expression of P2X4 receptor and suppressing ERK1/2 signaling.

In order to observe whether AST could specifically act on the P2X4 receptor, HEK293 cells transfected with P2X4 receptor plasmid were used. Our results showed that AST significantly inhibited the ATP-activated currents in HEK293 cells transfected with P2X4 receptor. The electrophysiological results combined with downregulation of P2X4 mRNA and protein after AST treatment confirmed that AST inhibited the transmission of nociceptive signaling by acting on the P2X4 receptor. Some literatures have shown that the expression of P2X4 is increased in inflammatory state (Winkelmann et al., 2019). After CCI injury, the rats were in the inflammatory state. Astragalin has an anti-inflammatory effect (Li et al., 2013; Fu et al., 2020). Therefore, the mechanism of astragalin in reducing the expression of P2X4 receptor may be through inhibiting inflammation. In this study, we also found that the expression of TNF-α receptor in CCI rats was decreased after administration of astragalin.

It has been reported that there is interaction between neurons and SGCs in the sensory ganglia (Huang et al., 2013), and purinergic receptors and inflammatory substances are involved in the communication (Hanani, 2012). Our supplementary experimental results showed that 5-BDBD could decrease P2X4 receptor expression and relieve pain behaviors in CCI rats, which suggested that P2X4 receptor activation plays an important role in neuropathologic pain behaviors caused by CCI. Therefore, astragalin may relieve the pain behaviors of CCI rats by reducing the upregulated expression and function of P2X4 receptor.

In conclusion, the upregulated P2X4 receptor in SGCs of dorsal root ganglion is involved in the process of CCI-induced neuropathic pain. AST reduces the upregulated expression and function of P2X4 receptor and then inhibits the activation of SGC, thus alleviating the pain behaviors of CCI rats. These results provide evidence that AST has therapeutic potential for the effective clinical management of neuropathic pain.

## Data Availability Statement

The original contributions presented in the study are included in the article/Supplementary Material, further inquiries can be directed to the corresponding author/s.

## Ethics Statement

The animal study was reviewed and approved by the Animal Protection and Use Committee of the Medical School of Nanchang University.

## Author Contributions

SDL and SML designed the study. MW, YW, and XC prepared the DRG tissue samples and performed immunofluorescence analysis, microscopy, and data analysis, and wrote the manuscript. MW, SZL, NW, and RS performed the pain behavior experiment. MW and SZL performed western blotting and qPCR experiments and data analysis. RS and JX assisted with animal breeding and maintenance. SML supervised the study and contributed to manuscript writing. All authors read and approved the final manuscript.

## Conflict of Interest

The authors declare that the research was conducted in the absence of any commercial or financial relationships that could be construed as a potential conflict of interest.

## Footnotes

1 http://autodock.scripps.edu/

2 http://mgltools.scripps.edu/

3 https://pymol.org/2/

## Abbreviations

ANOVA: analysis of variance
AST: astragalin
DRG: dorsal root ganglia
CCI: chronic constriction injury
DMSO: dimethyl sulfoxide
ERK: extracellular signal-regulated kinase
GFAP: glial fibrillary acidic protein
MWT: mechanical withdrawal threshold
P2X: purinergic receptor 2X
PBS: phosphate-buffered saline
pERK: phosphorylated extracellular-regulated protein kinase
SGC: satellite glial cell
TNF-R1: tumor necrosis factor receptor 1
TWL: thermal withdrawal latency.

## References

[B1] Burnstock G. (2013). Purinergic mechanisms and pain—An update. *Eur. J. Pharmacol.* 716 24–40. 10.1016/j.ejphar.2013.01.078 23524093

[B2] Burnstock G. (2017). Purinergic signalling and neurological diseases: an update. *CNS Neurol. Disord. Drug Targets* 16 257–265. 10.2174/1871527315666160922104848 27658510

[B3] Costa F. A. Moreira N. F. (2015). Satellite glial cells in sensory ganglia: its role in pain. *Braz. J. Anesthesiol.* 65 73–81. 10.1016/j.bjane.2013.07.013 25497752

[B4] Fitzgerald M. McKelvey R. (2016). Nerve injury and neuropathic pain - A question of age. *Exp. Neurol.* 275 296–302. 10.1016/j.expneurol.2015.07.013 26220898 PMC4691235

[B5] Fu R. Chen F. Guo Y. R. (2020). Anti-inflammatory mechanism and active ingredients of the Chinese tallow tree. *J. Ethnopharmacol.* 250:112497. 10.1016/j.jep.2019.112497 31870794

[B6] Gu N. Peng J. Murugan M. Wang X. Eyo U. B. Sun D. (2016). Spinal microgliosis due to resident microglial proliferation is required for pain hypersensitivity after peripheral nerve injury. *Cell Rep.* 16 605–614. 10.1016/j.celrep.2016.06.018 27373153 PMC4956495

[B7] Hanani M. (2005). Satellite glial cells in sensory ganglia: from form to function. *Brain Res. Rev.* 48 457–476. 10.1016/j.brainresrev.2004.09.001 15914252

[B8] Hanani M. (2012). Intercellular communication in sensory ganglia by purinergic receptors and gap junctions: implications for chronic pain. *Brain Res.* 1487 183–191. 10.1016/j.brainres.2012.03.070 22771859

[B9] Huang L. Gu Y. Chen Y. (2013). Communication between neuronal somata and satellite glial cells in sensory ganglia. *Glia* 61 1571–1581. 10.1002/glia.22541 23918214 PMC3758405

[B10] Jarvis M. F. (2010). The neural-glial purinergic receptor ensemble in chronic pain states. *Trends Neurosci.* 33 48–57. 10.1016/j.tins.2009.10.003 19914722

[B11] Jia T. Rao J. Zou L. Zhao S. Yi Z. Wu B. (2017). Nanoparticle-encapsulated curcumin inhibits diabetic neuropathic pain involving the P2Y12 receptor in the dorsal root ganglia. *Front. Neurosci.* 11:755. 10.3389/fnins.2017.00755 29422835 PMC5788895

[B12] Jurga A. M. Piotrowska A. Makuch W. Przewlocka B. Mika J. (2017). Blockade of P2X4 receptors inhibits Neuropathic pain-related behavior by preventing MMP-9 activation and, consequently, pronociceptive interleukin release in a rat model. *Front. Pharmacol.* 8:48. 10.3389/fphar.2017.00048 28275350 PMC5321202

[B13] Kobayashi K. Yamanaka H. Noguchi K. (2013). Expression of ATP receptors in the rat dorsal root ganglion and spinal cord. *Anatom. Sci. Intern.* 88 10–16. 10.1007/s12565-012-0163-9 23179910

[B14] Kushnir R. Cherkas P. S. Hanani M. (2011). Peripheral inflammation upregulates P2X receptor expression in satellite glial cells of mouse trigeminal ganglia: a calcium imaging study. *Neuropharmacology* 61 739–746. 10.1016/j.neuropharm.2011.05.019 21645532

[B15] Leung L. Cahill C. M. (2010). TNF-alpha and neuropathic pain–a review. *J. Neuroinflamm.* 7:27. 10.1186/1742-2094-7-27 20398373 PMC2861665

[B16] Li F. Y. Liang D. J. Yang Z. T. Wang T. C. Wang W. Song X. J. (2013). Astragalin suppresses inflammatory responses via down-regulation of NF-κB signaling pathway in lipopolysaccharide-induced mastitis in a murine model. *Int. Immunopharmacol.* 17 478–482. 10.1016/j.intimp.2013.07.010 23928506

[B17] Li L. Sheng X. Zhao S. Zou L. Han X. Gong Y. (2017). Nanoparticle-encapsulated emodin decreases diabetic neuropathic pain probably via a mechanism involving P2X3 receptor in the dorsal root ganglia. *Purinerg. Signal.* 13 559–568. 10.1007/s11302-017-9583-2 28840511 PMC5714846

[B18] Murai N. Sekizawa T. Gotoh T. Watabiki T. Takahashi M. Kakimoto S. (2016). Spontaneous and evoked pain-associated behaviors in a rat model of neuropathic pain respond differently to drugs with different mechanisms of action. *Pharmacol. Biochem. Behav.* 141 10–17. 10.1016/j.pbb.2015.11.008 26597514

[B19] Peng H. Zou L. Xie J. Wu H. Wu B. Zhu G. (2017). lncRNA NONRATT021972 siRNA decreases diabetic neuropathic pain mediated by the P2X3 receptor in dorsal root ganglia. *Mol. Neurobiol.* 54 511–523. 10.1007/s12035-015-9632-1 26742527

[B20] Popiolek-Barczyk K. Kolosowska N. Piotrowska A. Makuch W. Rojewska E. Jurga A. M. (2015). Parthenolide relieves pain and promotes M2 microglia/macrophage polarization in rat model of neuropathy. *Neural Plast.* 2015:676473. 10.1155/2015/676473 PMC4452088 26090236

[B21] Rey D. Miranda S. P. Alves F. T. Goncalves R. Silva F. M. Costa G. M. (2019). Astragalin augments basal calcium influx and insulin secretion in rat pancreatic islets. *Cell Calcium* 80 56–62. 10.1016/j.ceca.2019.03.009 30965223

[B22] Rojewska E. Piotrowska A. Makuch W. Przewlocka B. Mika J. (2016). Pharmacological kynurenine 3-monooxygenase enzyme inhibition signifificantly reduces neuropathic pain in a rat model. *Neuropharmacology* 102 80–91. 10.1016/j.neuropharm.2015.10.040 26524415

[B23] Rollini F. Franchi F. Angiolillo D. J. (2017). Switching P2Y12 receptor inhibiting therapies. *Intervent. Cardiol. Clin.* 6 67–89. 10.1016/j.iccl.2016.08.006 27886824

[B24] Spahr N. Hodkinson D. Jolly K. Williams S. Howard M. Thacker M. (2017). Distinguishing between nociceptive and neuropathic components in chronic low back pain using behavioural evaluation and sensory examination. *Musculoskel. Sci. Pract.* 27 40–48. 10.1016/j.msksp.2016.12.006 28637600 PMC5329124

[B25] Stokes L. Layhadi J. A. Bibic L. Dhuna K. Fountain S. J. (2017). P2X4 receptor function in the nervous system and current breakthroughs in pharmacology. *Front. Pharmacol.* 8:291. 10.3389/fphar.2017.00291 28588493 PMC5441391

[B26] Trang T. Salter M. W. (2012). P2X4 purinoceptor signaling in chronic pain. *Purinerg. Signal.* 8 621–628. 10.1007/s11302-012-9306-7 22528681 PMC3360095

[B27] Tsuda M. (2017). P2 receptors, microglial cytokines and chemokines, and neuropathic pain. *J. Neurosci. Res.* 95 1319–1329. 10.1002/jnr.23816 27376880

[B28] Tsuda M. Kuboyama K. Inoue T. Nagata K. Tozaki-Saitoh H. Inoue K. (2009b). Behavioral phenotypes of mice lacking purinergic P2X4 receptors in acute and chronic pain assays. *Mole. Pain* 5:28. 10.1186/1744-8069-5-28 19515262 PMC2704200

[B29] Tsuda M. Toyomitsu E. Kometani M. Tozaki-Saitoh H. Inoue K. (2009a). Mechanisms underlying fibronectin-induced up-regulation of P2X4R expression in microglia: distinct roles of PI3K-Akt and MEK-ERK signalling pathways. *J. Cell Mol. Med.* 13 3251–3259. 10.1111/j.1582-4934.2009.00719.x 19298529 PMC4516482

[B30] Tsuda M. Masuda T. Tozaki-Saitoh H. Inoue K. (2013). P2X4 receptors and neuropathic pain. *Front. Cell. Neurosci.* 7:191. 10.3389/fncel.2013.00191 24191146 PMC3808787

[B31] Tsuda M. Shigemoto-Mogami Y. Koizumi S. Mizokoshi A. Kohsaka S. Salter M. W. (2003). P2X4 receptors induced in spinal microglia gate tactile allodynia after nerve injury. *Nature* 424 778–783. 10.1038/nature01786 12917686

[B32] Ulmann L. Hatcher J. P. Hughes J. P. Chaumont S. Green P. J. Conquet F. (2008). Up-regulation of P2X4 receptors in spinal microglia after peripheral nerve injury mediates BDNF release and neuropathic pain. *J. Neurosci.* 28 11263–11268. 10.1523/jneurosci.2308-08.2008 18971468 PMC6671487

[B33] Wang S. Xu H. Zou L. Xie J. Wu H. Wu B. (2016). LncRNA uc.48+ is involved in diabetic neuropathic pain mediated by the P2X3 receptor in the dorsal root ganglia. *Purinerg. Signal.* 12 139–148. 10.1007/s11302-015-9488-x 26686228 PMC4749536

[B34] Winkelmann V. E. Thompson K. E. Neuland K. Jaramillo A. M. Fois G. Schmidt H. (2019). Inflammation-induced upregulation of P2X4 expression augments mucin secretion in airway epithelia. *Am. J. Physiol. Lung. Cell Mol. Physiol.* 316 L58–L70. 30358443 10.1152/ajplung.00157.2018 PMC6883286

[B35] Yang R. Li L. Yuan H. Liu H. Gong Y. Zou L. (2019). Quercetin relieved diabetic neuropathic pain by inhibiting upregulated P2X4 receptor in dorsal root ganglia. *J. Cell. Physiol.* 234 2756–2764. 10.1002/jcp.27091 30145789

[B36] Ying M. Liu H. Zhang T. Jiang C. Gong Y. Wu B. (2017). Effect of artemisinin on neuropathic pain mediated by P2X4 receptor in dorsal root ganglia. *Neurochem. Intern.* 108 27–33. 10.1016/j.neuint.2017.02.004 28192150

[B37] Yuan H. Ouyang S. Yang R. Li S. Gong Y. Zou L. (2018). Osthole alleviated diabetic neuropathic pain mediated by the P2X4 receptor in dorsal root ganglia. *Brain Res. Bull.* 142 289–296. 10.1016/j.brainresbull.2018.08.008 30118750

[B38] Zhao L. Li D. Liu N. Liu L. Zhang Z. Gao C. (2018). Correlation of TGN-020 with the analgesic effects via ERK pathway activation after chronic constriction injury. *Mol. Pain* 14:1744806918796057. 10.1177/1744806918796057 PMC6113736 30152258

[B39] Zheng D. H. Liu D. W. Liu N. Kuang Y. K. Tai Q. (2019). Astragalin reduces lipopolysaccharide-induced acute lung injury in rats via induction of heme oxygenase-1. *Archiv. Pharm. Res.* 42 704–711. 10.1007/s12272-019-01171-8 31250343

