# Supplementary figures and images for: Astragalin Alleviates Neuropathic Pain by Suppressing P2X4-Mediated Signaling in the Dorsal Root Ganglia of Rats
Source: Front Neurosci. 2021 Jan 11;14:570831. doi: 10.3389/fnins.2020.570831 (PMC7829479; doi:10.3389/fnins.2020.570831)

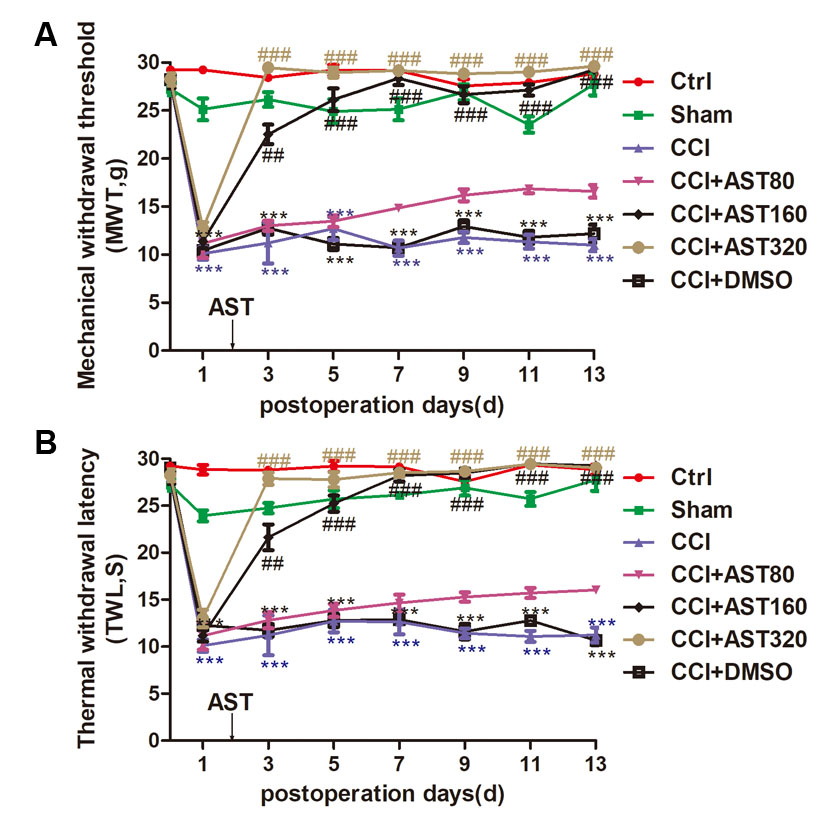

Supplement: Supplementary file 1 [file Image_1.TIF]

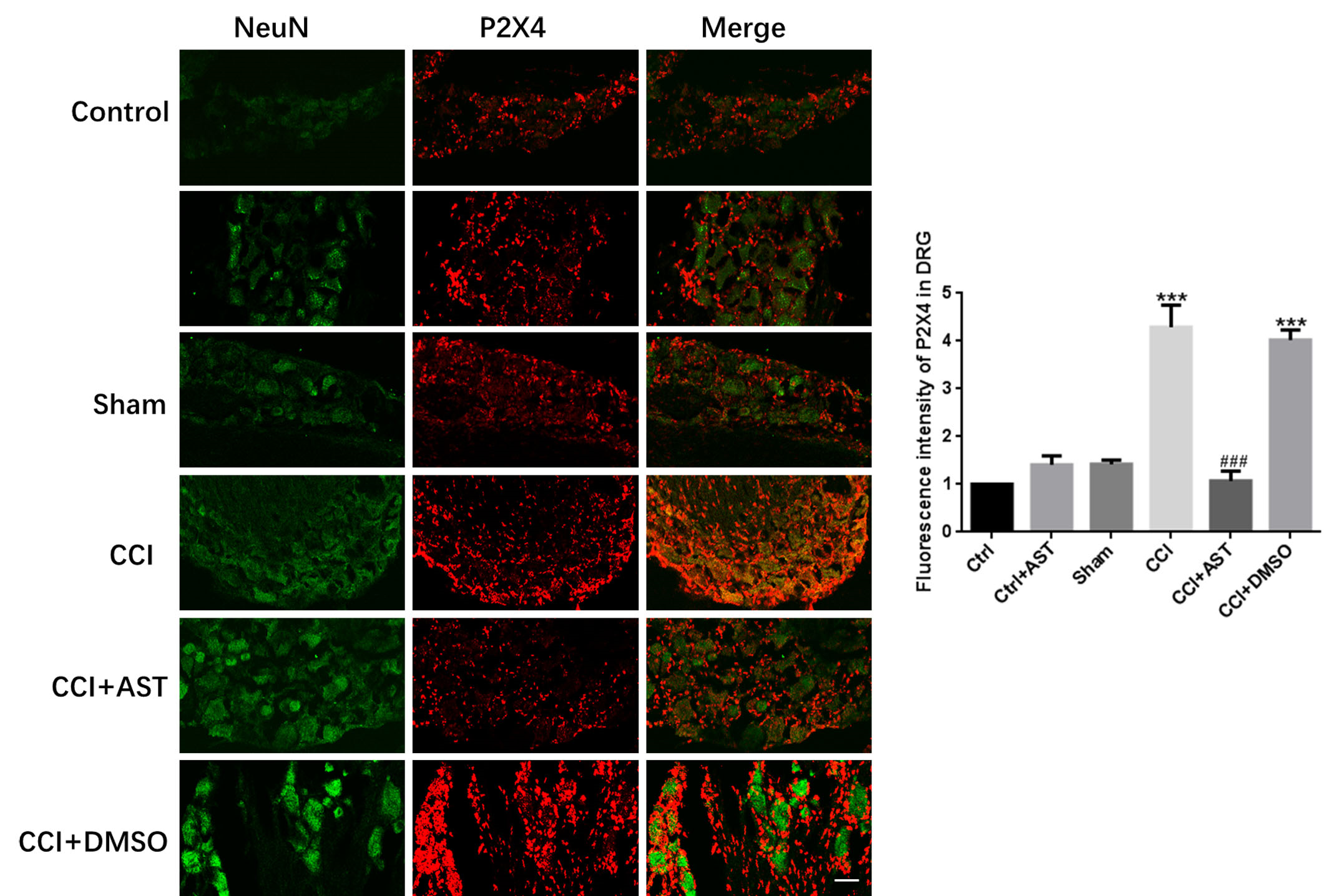

Supplement: Supplementary file 2 [file Image_2.TIF]
